# Experimental Investigation into Dissociation Characteristics of Methane Hydrate in Sediments with Different Contents of Montmorillonite Clay

**DOI:** 10.1021/cbe.4c00174

**Published:** 2025-03-03

**Authors:** Chang Chen, Yu Zhang, Xiaosen Li, Yuru Chen, Du Wang

**Affiliations:** † Key Laboratory of Gas Hydrate, Guangzhou Institute of Energy Conversion, Chinese Academy of Sciences, Guangzhou 510640, P. R China; ‡ University of Science and Technology of China, Hefei 230026, P. R China; § Guangdong Provincial Key Laboratory of New and Renewable Energy Research and Development, Chinese Academy of Sciences, Guangzhou 510640, P. R. China

**Keywords:** Methane hydrate, Montmorillonite clay, Depressurization, Dissociation rate, Temperature distribution, Electrical resistance

## Abstract

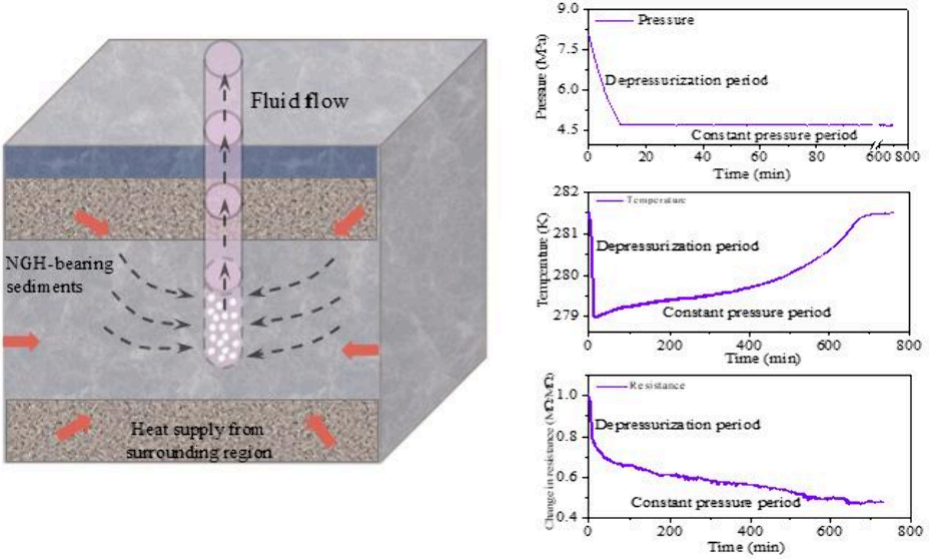

The characteristics of gas production in sediments are crucial to the safe and efficient exploitation of gas hydrate resources. However, research on methane hydrate dissociation in these sediments, particularly in silty-clayey sediments, which are commonly found in nature, remains limited and contains significant gaps. To address this, a series of depressurization experiments were conducted to investigate the dissociation behavior of methane hydrate in silty-clayey sediments with montmorillonite contents ranging from 0 to 20 wt %. The results indicate that montmorillonite significantly inhibits methane hydrate dissociation. When the montmorillonite content increases from 10 to 20 wt %, the average dissociation rate of methane hydrate decreases by approximately 47%–78% compared to sandy sediments. An excess temperature drop of around 0.13 to 0.40 K was observed in the depressurization process as the montmorillonite content increased from 10 to 20 wt %. Methane hydrate dissociates unevenly in montmorillonite clay-bearing sediments due to the nonuniform distribution of the methane hydrate, coupled with the low thermal conductivity and high-water absorption capacity of montmorillonite, which restrict the supply of extra heat. The electrical resistance changes further reveal that the increased bound water content in clayey sediments reduces the impact of water fluctuation on the resistivity changes. Consequently, the resistivity changes in sandy sediments are more pronounced compared to silty-clayey sediments. These findings provide valuable insights for optimizing methane hydrate production technology via depressurization.

## 1 Introduction

As global energy demand continues to rise and climate change worsens, reducing greenhouse gas emissions and developing clean energy have become priority concerns for the international community. Natural gas hydrate (NGH), also known as methane hydrate (MH), is an ice-like crystalline compound formed under low-temperature and high-pressure conditions when methane (CH_4_) molecules are encased by water molecules through hydrogen bonding.^1,2^ NGHs are widely distributed in marine sediments and permafrost regions, especially in sediments along continental slopes and rises at water depths exceeding 300 m.^2,3^ Global reserves of NGHs are estimated to be immense, with conservative estimates reaching 1.5 × 10^16^ m^3^, likely surpassing traditional natural gas reserves.^4,5^ The substantial resource potential and relatively low carbon emissions of NGHs position them as a promising strategic energy source for the future.^6^

In recent years, several countries such as Canada, the United States, Japan, and China have carried out various short-term pilot projects for NGH extraction,^3^ employing methods like thermal stimulation, depressurization, inhibitor injection, CO_2_ replacement, and combined depressurization.^7−10^ Among these methods, depressurization (single or multistage) is widely regarded as the most efficient and cost-effective extraction method.^11,12^ However, commercial exploitation of NGH faces significant technical and economic challenges. The gas release stages result in a temperature drop in the reservoir, which subsequently affects the dissociation front and gas production rate of MH.^13^ As MH dissociate and water–gas flow through the reservoir, the dissociation rate gradually decreases, driven by the efficiency of heat and mass transfer.^14,15^ Therefore, comprehensive research on the dissociation characteristics of MH-bearing sediments and the associated heat and mass transfer behaviors during depressurization is crucial for optimizing extraction methods and enhancing the gas production efficiency.

To date, extensive research using experimental simulations and numerical modeling has investigated MH dissociation behavior in various sediment media, primarily quartz sand and glass beads.^16−21^ These studies primarily focused on the effects of water distribution, pore size distribution, particle size, and porosity on the dissociation rate and heat and mass transfer behavior of MH, aiming to evaluate the feasibility and efficiency of extraction methods, such as depressurization, thermal stimulation, and CO_2_ replacement. Furthermore, studies have analyzed production dynamics in terms of gas production, recovery efficiency, heat transfer, and fluid flow,^22,23^ while optimizing production strategies (e.g., depressurization rate, magnitude, and mode) to improve extraction efficiency.^13,24−26^ However, actual NGH-bearing reservoirs exhibit significant variability and can be categorized into different types: (1) Sandstone-dominated reservoirs with high porosity and permeability (100–1000 mD), such as those in Alaska’s North Slope and Canada’s Mackenzie Delta;^27^ (2) coarse sand and silt-dominated reservoirs with lower permeability compared to sandstone (47–840 mD), such as the Green Canyon in the Gulf of Mexico and the Nankai Trough in Japan;^28^ (3) muddy silt-dominated reservoirs with low porosity and extremely low permeability (2–5 mD), such as those in the Shenhu area of the South China Sea.^3,29^ Core analyses of NGH test production areas in the South China Sea^29^ reveal that sediments are rich in silt and clay, with clay minerals primarily comprising montmorillonite (MMT), Illite, and minor kaolinite, with a mass fraction of 26–30%.^30^ Compared to sandy minerals, clay has smaller particle sizes, larger specific surface areas, higher surface activity, lower thermal conductivity and permeability, as well as a unique layered brick structure.^31−33^ Among common clays, MMT exhibits the strongest surface and interlayer water absorption, along with significant swelling characteristics and low permeability.^31^ In the depressurization production period, the low thermal conductivity, low permeability, and strong water-absorbing swelling properties of MMT may significantly influence MH dissociation and gas production efficiency.^34−38^ Currently, research on MH dissociation characteristics in clay sediments has mainly focused on MH dissociation and distribution in sandy sediments, while research on other types of clay is limited.^9,32^ Therefore, understanding the dissociation mechanisms of MH by depressurization in MMT-rich systems based on the unique physical properties of MMT, is critical for optimizing extraction methods and designing safe and efficient production strategies.

In summary, clay minerals, particularly MMT, significantly influence MH dissociation conditions, distribution, and reservoir heat and mass transfer properties by tuning the pore structure and water distribution in sediments. Although some studies^34,39,40^ have explored the effects of clay minerals on MH dissociation conditions and MH formation kinetics, systematic research on MH dissociation characteristics, gas production behavior, and heat transfer properties of silty-clayey sediments remains limited, with a lack of detailed quantitative analysis. Given the critical role of clay minerals in the efficient exploitation of NGH reservoirs, it is essential to elucidate their impact on the MH dissociation process by depressurization. Due to the high costs and unavoidable disturbances during the recovery and transportation of in situ MH-bearing silty-clayey sediments, preparing representative MH-bearing silty-clayey sediment in the laboratory has become a necessary research approach. In this study, based on the compositional characteristics of sediments in the South China Sea, sediments with varying clay-to-silt ratios were prepared by uniformly mixing quartz sand and MMT. The dissociation characteristics of MH in these sediments were systematically investigated using the depressurization method. This study focused on analyzing the effects of varying the MMT content on gas production rates during depressurization. Additionally, variations in temperature and electrical resistance within sediment layers were examined to elucidate the influence of the MMT content on heat transfer and gas release behavior during depressurization.

## 2 Experimental Section

### 2.1 Apparatus

**Figure 1:**
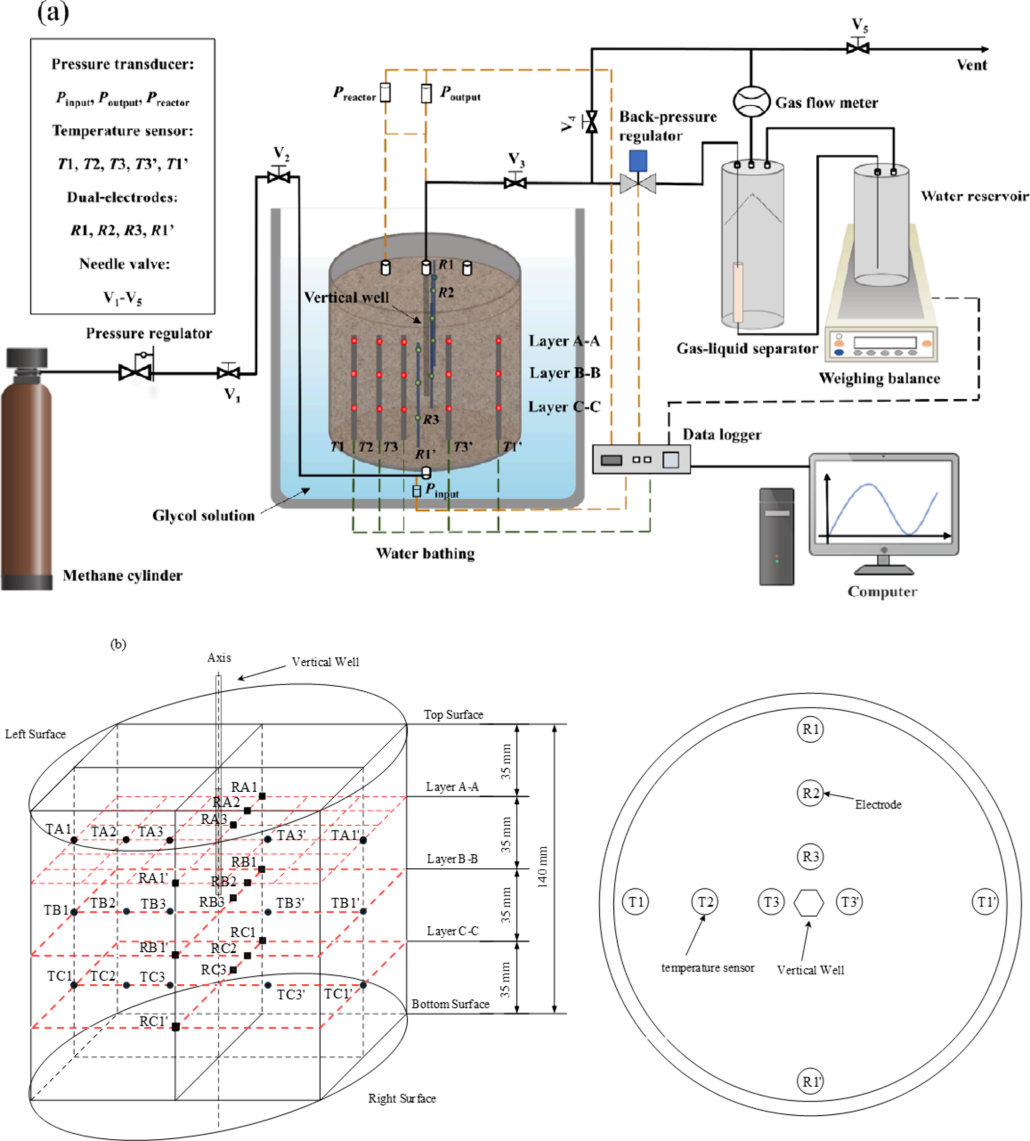
(a) Schematic of the experimental apparatus. (b) Distributions of temperature measuring points and electrical resistance measuring points of each layer in the reactor. Reproduced with permission from ref (41). Copyright 2024 Elsevier.

The experimental apparatus for MH formation and dissociation is shown in Figure 1. This apparatus mainly consists of three parts: a high-pressure reactor with a water bath, a production control system, and a data acquisition system. The high-pressure reactor has a volume of approximately 2.5 L, with an inner diameter of 150 mm and a height of 140 mm. The pressure limitation is designed up to 22 MPa. The water bath (XT5204-BS30, Xutemp Temptech Co., Ltd., China) maintains a temperature range of 243.15–368.15 K with an accuracy of ±0.1 K. The production control system includes a PID controller (TESCOM, accuracy of ±0.1 MPa, 0–25 MPa) and a gas–liquid separator coupled with an electronic balance (Sartorius BSA6202S). Gas production is recorded by a flowmeter (Changchun Alpha Instrument Co., Ltd., China, precision of ±10 mL/min). To monitor the experimental data in the reactor and production unit, three pressure transducers (accuracy of 0.1% FS, 0–25 MPa), four three-point multipoint dual-electrodes (a pair of electrodes), and five three-point Pt100 thermocouples (accuracy of ±0.1 K), are installed. Three pressure transducers are connected to the production well (*P*_reactor_), gas input (*P*_input_), and gas output (*P*_output_) of the reactor. All the pressure and temperature data are recorded by a data acquisition system every 20 s and stored on the experimental computer. In this study, as the trends of *P*_reactor_, *P*_input_ and *P*_output_ remained consistent throughout MH formation and dissociation, all references to pressure in the following sections refer to *P*_reactor_.

The distribution of the thermocouples (15 measuring points) and dual-electrodes (12 measuring points) in the reactor is shown in Figure 1(b). Within the reactor, thermocouples are spaced 35 mm apart, dividing the interior into three layers: the upper (A-A), middle (B–B), and bottom (C–C). Each layer consists of 5 temperature sensors and 4 pairs of electrodes. From top to bottom layer, the thermal couples in each layer are labeled as TA1, TA2, TA3, TA3′, TA1’... TC3′, TC1’. TA1’ and TA3′ had the same distance to the center of the reactor as TA1 and TA3, respectively. TA1, TB1, and TC1 were located close to the inner wall of the reactor, and TA3, TB3, and TC3 were located near the center of the reactor. As shown in Figure 1(b), taking an example of layer A-A, in which the temperature sensors (TA1, TA2, TA3) are installed at different radial positions (r = 10 mm, 37.5 mm, 65 mm). Similarly, the electrodes (RA1, RA2, RA3) are installed at different radial positions (r = 10 mm, 37.5 mm, 65 mm), and RA1’ has the same distance to the center of the reactor as RA1. Additionally, as shown in Figure 1(b), the vertical well in the center of the reactor is used as the production well. The production well consists of a vertical stainless-steel tube with a diameter of 4.0 mm. To prevent clogging at the wellhead and ensure efficient fluid flow, multiple small grooves are evenly distributed and uniformly distributed around the circumference of the production well, approximately 80 mm below the top of the reactor. The outer surface of the production well is wrapped with two layers of 300-mesh screens, allowing fluid to flow upward through these grooved areas. The experimental system has also been described in detail in our previous study.^41^

### 2.2 Materials

CH_4_ gas (99.95% purity, supplied by Yingde Xizhou Gas Co., Ltd., China) and deionized water were used for MH formation in this study. Quartz sand and MMT clay (supplied by Shanghai Macklin Biochemical Technology Co., Ltd., China) with densities of 2.50 and 2.60 g/cm^3^, respectively, were measured using an ASIQACIV200–2 instrument with N_2_ as the adsorbate. The median particle size of sand and MMT is 145.65 and 12.43 μm, respectively, determined from three tests using a Mastersizer 2000E laser micrometer particle size analyzer (Malvern Instruments, UK).

### 2.3 Procedures

In this work, MH-bearing sediments were synthesized in sandy and silty-clayey sediments using the gas-saturated method.^41,42^ The procedures for preparing the silica sand/clay mixture samples: First, various mass fractions of silica sand and clay were mixed in a glass dish. After achieving through mixing of the sand/clay mixture, a certain amount of water was added to the dry sediments to achieve the desired water content. A brief description of the experimental procedures for MH formation and dissociation in silty-clayey sediment is summarized as follows: (a) Approximately 3200–3300 g of a premixed wet sand/clay mixture was packed layer-by-layer into the reactor. (b) Sealing and submerging the reactor in a constant temperature water bath with a stable ambient temperature of 293.15 K. (c) CH_4_ gas was injected to remove the residual air, and the reactor was pressurized to the experimental pressure of 18.50 MPa. (d) MH formation was induced by cooling and maintaining the reactor temperature to 281.35 K. (e) MH formation was deemed to be complete after the inlet and outlet pressures reached the target pressure. (f) A Back-pressure regulator was set to the desired pressure value to prepare for depressurization experiments (g) The reactor pressure was gradually reduced (at about 0.8–1.0 L/min) to 4.70 by releasing CH_4_ gas and maintained within the range of 4.70–4.75 MPa. (h) The gas production process was considered complete when gas release diminished significantly. Detailed experimental conditions, such as initial pressure (*P*_0_), mass of MMT (M_MMT_), sand (M_sand_) and three phase saturations are summarized in Table 1.

**Table 1:** Summary of Experimental Conditions Used in All of the Experimental Cases

Exp. No.	Case 1	Case 2	Case 3	Case 4
Initial pressure (MPa)	8.10	8.22	8.16	8.07
Mass of sand, M_sand_ (g)	3126	2905	2710	2530
Mass of MMT, M_MMT_ (g)	0	326	480	635
Clay content (wt %)	0	10	15	20
Initial saturation of MH, *S*_H_ (%)	37.22	36.61	36.21	37.13
Initial saturation of water, *S*_w_ (%)	19.61	20.42	20.10	19.07
Initial saturation of gas, *S*_g_ (%)	43.17	42.97	43.68	43.80
Producton pressure (MPa)	4.70	4.70	4.70	4.70
Production temperature (K)	281.41	281.55	281.63	281.66

### 2.4 Calculations

The calculation method for phase saturations of water saturation (*S*_w_), gas saturation (*S*_g_), and gas hydrate saturation (*S*_H_) in the sediments after MH formation was followed by Li et al.^9,13,41^ The equations for quantifying the amount of MH in sediments during the MH formation and dissociation processes are summarized as follows:

The reaction of MH formation is described as
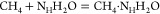
1

It is assumed that the pore volume of the reactor *V*_pore_ (cm^3^) remained constant before and after MH formation, and the relationships of the volumes of water, gas and hydrate are as follows:
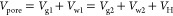
2
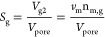
3
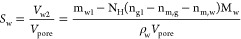
4
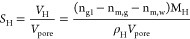
5
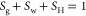
6where *V*_g1_ and *V*_w1_ are the volumes (cm^3^) of gas and water before MH formation, respectively. *V*_g2_, *V*_w2_ and *V*_H_ are the volumes (cm^3^) of gas, water, and gas hydrate after MH formation, respectively. *v*_m_ is the molar volume of CH_4_ gas (m^3^/mol), which can be calculated by the Peng–Robinson equation^43^ at different time. n_g1,_ and n_m,g_, are the amounts of gas (mol) before and after MH formation. n_m,w_ is the amount of dissolved gas (mol) in the aqueous phase. m_w1_ is the total mass of water (g) injected into the reactor. *M*_W_ and *M*_H_ represent the molar amounts of water and MH (g/mol), respectively. ρ_w_ and ρ_H_ represent the density of water and MH (g/cm^3^), respectively. *V*_pore_ is the pore volume of the reactor (cm^3^).

Due to the limited solubility of CH_4_ gas, it is assumed that no CH_4_ gas is dissolved in the aqueous phase. The hydration number (N_H_) is assumed to be at an ideal stoichiometric value of 5.75. The molar mass of water and MH is 18 g/mol and 119.5 g/mol, respectively. The density of MH is 0.94 g/cm.^39,41^

During the MH dissociation process by depressurization, the reactor pressure is maintained within the range of 4.70–4.75 MPa, allowing excess gas to flow through a control valve. The real-time volume of dissociated CH_4_ gas, representing the increase in total gas volume within the system, is calculated as follows:^9^
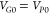
7
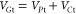
8
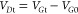
9where *V*_*G*__0_ and *V*_*P*__0_ are the volumes (cm^3^) of gas in the system and in the pore at the beginning of depressurization, respectively. *V*_*G*__t_ is the total volume (cm^3^) of gas in the system at time t. *V*_*P*__t_ is the volumes (cm^3^) of gas in the pore at time t. *V*_*C*__t_ is the volume (cm^3^) of gas flowing through the gas flow meter at time t. *V*_*D*__t_ is the volume of dissociated gas at time t.

The gas recovery ratio φ is calculated by the following equation:
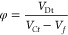
10where *V*_*f*_ is the total volume (L) of free gas.

## 3 Results and Discussion

### 3.1 MH Dissociation Characteristics in Silty-Clayey Sediments by Depressurization

#### 3.1.1 Evolution of Pressure and Temperature

**Figure 2:**
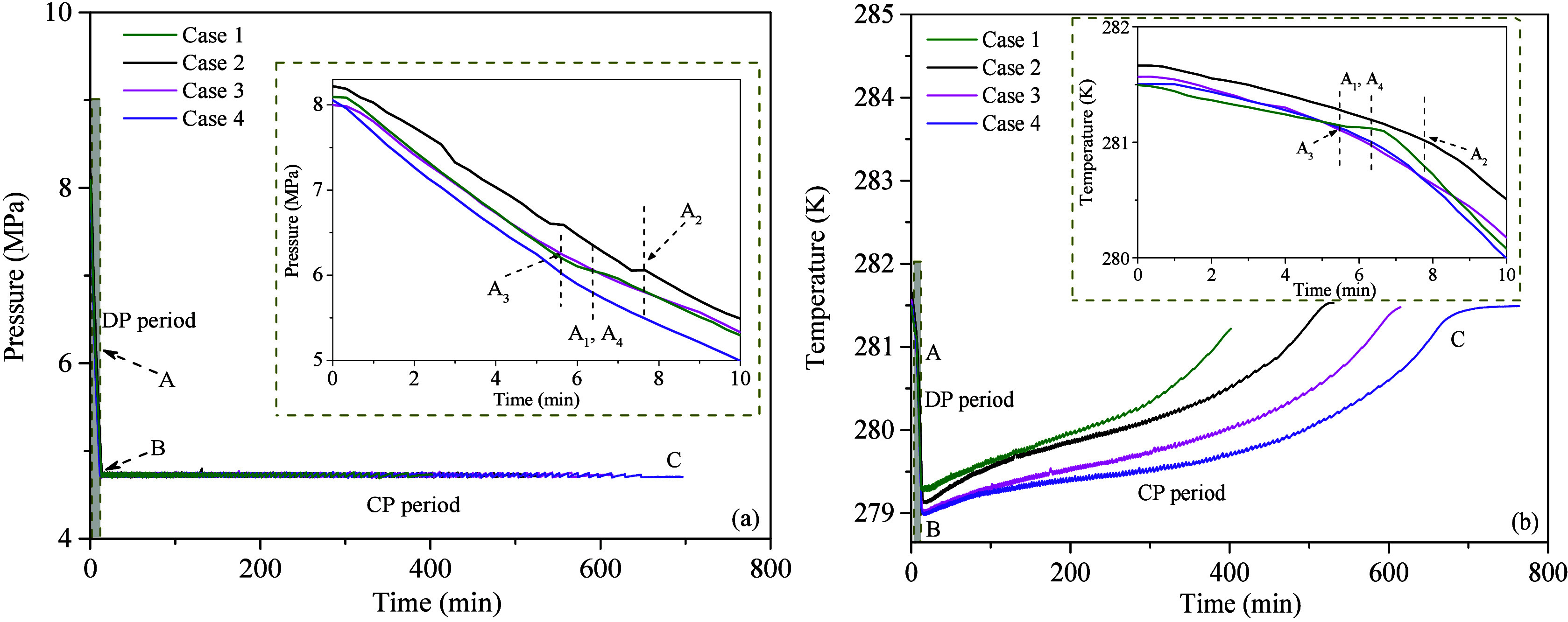
Variations of (a) pressure and (b) temperature over time in experiment Cases 1–4.

Figure 2(a) and (b) shows the changes in system pressure and average temperature during MH dissociation by depressurization for Cases 1–4. Time zero is the initial time of depressurization. From Figure 2 and the partially enlarged figure, it is evident that the pressure and temperature changes can be divided into two periods, the depressurization (DP) period and the constant-pressure (CP) period. The inflection points A_1_-A_4_ represent the initiation of MH dissociation for Cases 1–4, respectively. These points are determined by calculating the total gas increment in the system, as described in eqs 7–9, using a method similar to previous calculations.^9^ The points B_1_–B_4_ represent when the system pressures decrease to the predetermined production pressure of 4.70 MPa for Cases 1–4, respectively. Taking Case 1 as an example, during the DP period, after 7 min (point A_1_), the MH begins to dissociate. This is primarily indicated by a decrease in the rate of pressure decline and a significant increase in the rate of the temperature drop within the reactor. As the experiment continues, the pressure in the reactor rapidly decreases from about 8.10 to 4.70 MPa, and the temperature drops quickly from 281.50 K to around 279.25 K (point B_1_). This indicates that a significant amount of the inherent latent heat of the sediment is consumed during the initial stage of MH dissociation. In the CP period, as the MH continues to dissociate, the temperature within the reactor gradually increases due to a decrease in ambient heat transfer and a reduction in the driving force for MH dissociation. Once the MH dissociation is complete, the temperature increase ceases and the system stabilizes at around 281.40 K (point C). It should be noted that compared to Case 1, the inflection points in Cases 2–4 are less distinct. This may be due to the MH dissociation in clay-bearing sediments being a multistage process. Additionally, slight fluctuations in valve control during the early stages of the experiment may introduce minor errors into calculating these inflection points.

During the DP period, the average temperature change trends in Cases 2–4 are similar to those in Case 1, as shown in Figure 2(b). The difference is that the lowest temperature at the end of the DP period (point B) varies due to the influence of the MMT content. This indicates that compared to the sandy sediments, the dissociation temperature of MH in MMT clay-bearing sediments is lower under the same production pressure. This is mainly due to the fact that in the early period of MH dissociation, MH formed by strongly bound water dissociates first, followed by that formed by weakly bound water and free water.^39^ The equilibrium dissociation conditions for MH formed by strongly bound water are relatively stringent, leading to a more pronounced cooling effect in systems with a higher MMT content. In the CP period, different MMT contents have a significant impact on the rate of the temperature rise. The temperature increase rates of different cases are generally similar during the first 20 min. However, as the MH dissociates, the rate of temperature increase decreases with increasing MMT content, indicating that as MMT content increases, the heat transfer rate between the sediment and the surrounding environment decreases. At the end of the CP period, the duration for Cases 2–4 is extended by approximately 1.30, 1.50, and 1.75 times compared to Case 1, respectively, indicating that even at MMT contents below 20 wt %, heat transfer in the reservoir can be significantly hindered.

#### 3.1.2 Evolution of Gas Production

**Figure 3:**
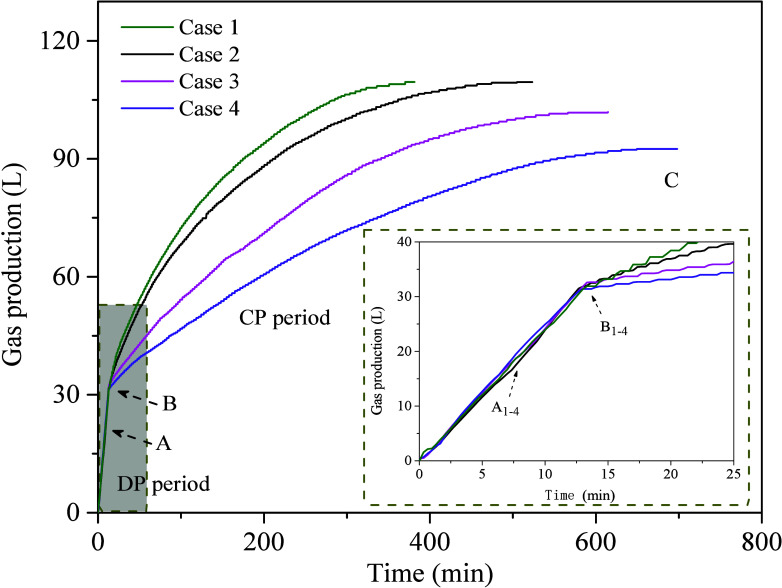
Variation in gas production over time in experiment Cases 1–4.

Figure 3 shows the cumulative gas production profiles during the gas production processes for each case. The cumulative gas production includes free gas and dissociated gas. Relevant results for gas production and the average MH dissociation rate in various experimental cases are summarized in Table 2. As shown in Figure 3, before point A, the produced gas is free gas and its production rate remains basically constant. After point A, the gas produced from dissociated MH begins to mix with the free gas. Due to the limitation in the gas production rate, the free gas production rate gradually decreases slightly (AB period). This observation aligns with experimental results from Nair et al. and Dong et al.^24,44^ From Figure 3 and Table 2, it is evident that the cumulative gas production difference among the different cases is relatively small, while the production of dissociated gas increases as the MMT content increases. During the CP period, the duration of cumulative gas production in different cases gradually increases. Compared with the sandy sediments and MMT clay-bearing sediments, increasing the MMT content from 10 to 20 wt %, results in a decrease in cumulative gas production by 0.1% to 16%. In other words, lower MMT content leads to higher CH_4_ recovery. Taking Case 1 and Case 3 as examples, the gas production at 13.3 and 13.5 min (point B) is 33.2 and 32.65 L, respectively, with gas produced from MH dissociation accounting for 8.33 and 12.28 L, respectively. By the end of CP period (Point C), the cumulative gas production in Case 1 and Case 3 reaches 109.63 and 101.80 L, respectively.

**Table 2:** Experimental Results in All of the Experimental Cases

Exp. No.	Cumulative gas production volume in DP period (L)	MH dissociation volume in DP period (L)	Cumulative gas production volume in CP period (L)	Cumulative gas production volume (L)	Average MH dissociation rate (L/min)	Average gas production rate (L/min)
Case 1	33.20	8.33	76.43	109.63	0.21	0.30
Case 2	31.51	9.56	77.01	108.52	0.11	0.16
Case 3	32.65	12.28	69.15	101.80	0.06	0.13
Case 4	31.41	14.09	61.12	92.53	0.05	0.12

**Figure 4:**
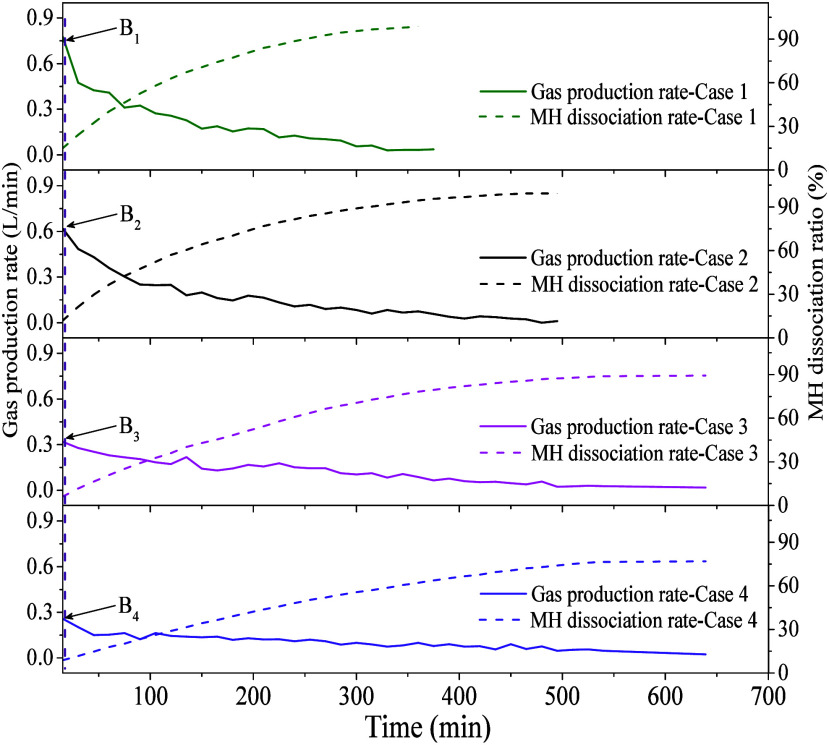
Evolution of MH dissociation rate during the CP period in experiment Cases 1–4.

The cumulative gas production results indicate that during the DP period gas production mainly derives from free gas. In contrast, the dissociated gas produced in the CP period accounts for more than 90% of the total gas production (see Table 2). Therefore, analyzing changes in the dissociation rate of MH during the CP process is crucial for assessing the impact of the MMT clay content on MH dissociation. Figure 4 shows changes in the gas production rate and gas recovery ratio over time for Cases 1–4 during the CP period. The dashed line represents the moment when the CP period begins (point B). As shown in Figure 4, in the early CP period, the gas production rates of different cases are relatively high, but the phenomenon of rapid gas production usually lasts only a short time, followed by a significant decrease. Taking Case 4 as an example, the gas production rate during the first 15 min is approximately 0.26 L/min. By 180 min, when the gas recovery ratio reaches 39.04%, the gas production rate drops rapidly to 45.55% of its initial value. As the dissociation of MH continues, the gas production rate in Case 4 decreases further, reaching 22.43% of its initial value when the gas recovery ratio reaches 70.57% at 450 min. Compared with different cases, the gas production rate in the MMT-bearing system consistently remains lower than that of Case 1 throughout the entire CP period. This trend correlates strongly with variations in temperature and pressure, indicating a good correlation between the rate of MH dissociation and changes in the reservoir temperature and heat transfer.

**Figure 5:**
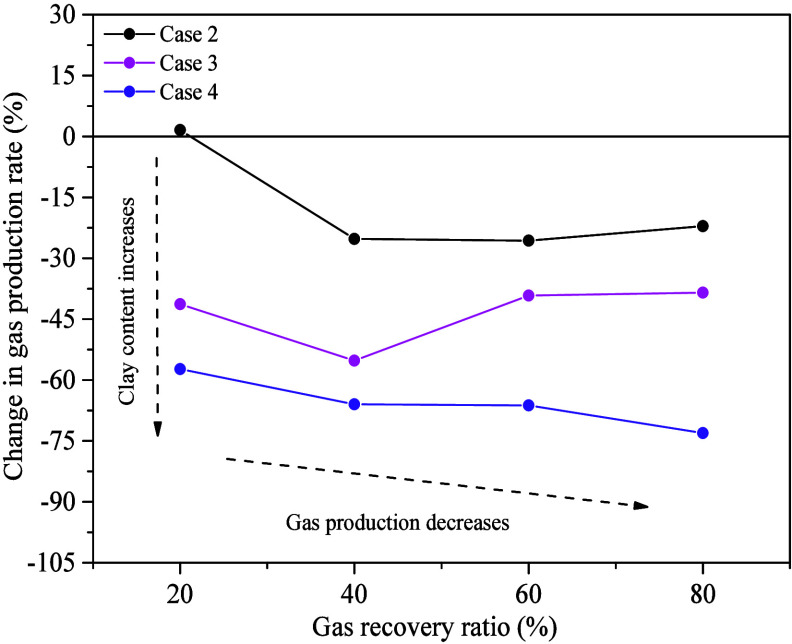
Comparison of the gas production rates between experiment Cases 2–4 and Case 1 at different gas recovery ratios in the CP period.

To quantify the influence of MMT on the MH dissociation rate, the percentage differences in the MH dissociation rate between Cases 2–4 and Case 1 at gas recovery ratios of 20%, 40%, 60%, and 80% (i.e., GR20, GR40, GR60, and GR80 stages), respectively. These stages correspond to the CP period during the gas production process, allowing for a more accurate comparison of the dissociation rates across different experimental cases. Figure 5 shows a comparison of the percentage differences in gas production rates among Cases 2–4 and Case 1 at different gas recovery ratios. In systems with high MMT content, the decrease in the gas production rate is more pronounced at different gas recovery stages. Specifically, in Case 1, when the gas recovery rates are 20%, 40%, 60%, and 80%, the gas production rates are 0.47 0.41, 0.26, and 0.17 L/min, respectively. Compared to the GR20 stage, the gas production rate in Case 1 decreases by 14%, 46%, and 63% in the GR40, GR60, and GR80 stages, respectively, with the rate in the GR80 stage being only 37% of that in the GR20 stage. In Case 4, the gas production rates in the GR40, GR60, and GR80 stages decrease by 31%, 57%, and 77% compared to the GR20 stage, respectively. Although Case 4 shows a greater decrease in gas production rate at each stage compared to Case 1, the overall trend indicates that systems with higher MMT content experience a more significant reduction in the gas production rate in the later stages, with the rate in the GR80 stage being only 23% of that in the GR20 stage. Analysis of the gas production rates of different experiments reveals that compared to Case 1, Case 4 shows a faster decline in gas production rate, with a decrease of over 55% across gas recovery ratios from 20% to 80%. Case 3 follows, with a decrease exceeding 35%, and Case 1 has a smaller decrease of nearly 20%. Notably, at GR20, the gas production rate for Case 2 is slightly higher than that of Case 1 by 1.58%. Furthermore, at GR40 and GR60, the gas production rates for Cases 3 and 4 are slightly higher than those at GR20. These slight fluctuations in gas production rates may be attributed to the dissociation kinetics of MH in the MMT clay-bearing sediments.

### 3.2 Analysis of Heat Transfer

**Figure 6:**
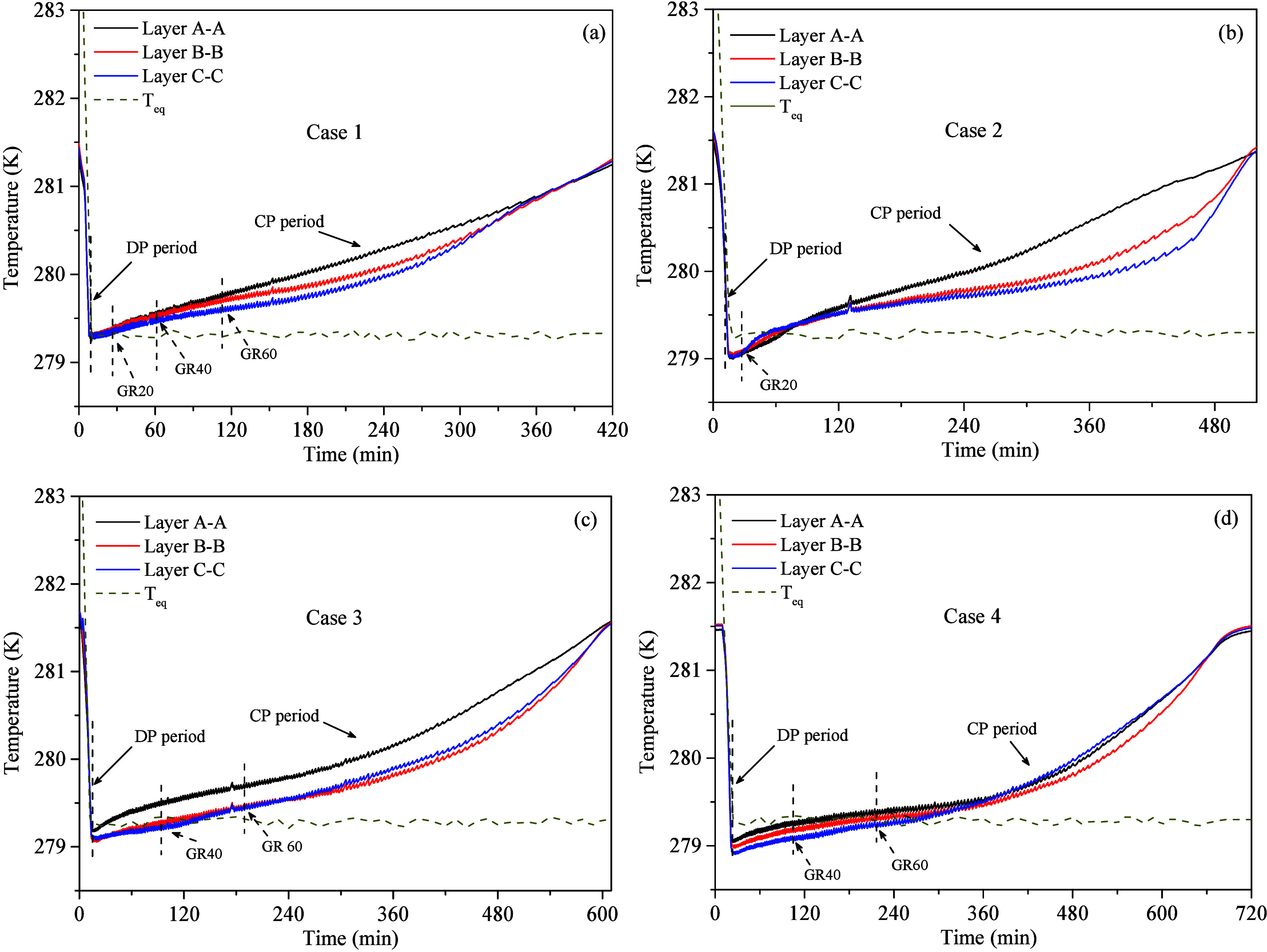
Evolution of average temperature at layers A-A, B–B, and C–C in experiment Cases 1–4.

The dissociation of MH by depressurization in sediments involves complex phase changes, heat conduction, mass transfer, and multiphase seepage processes. Previous studies^34,39^ have shown that clay significantly affects the MH phase equilibrium conditions, the MH distribution, and the thermal conductivity and the permeability of the reservoir. Additionally, Sun et al.^47^ demonstrated that gas release in heterogeneous sediments can increase pore pressure, potentially altering pore network connectivity and effective permeability. In this work, due to the limitation of the reactor size, there is little discrepancy in the pressures at different measuring points during both the DP and CP periods, which is consistent with previous studies.^9,42^ Consequently, issues related to heterogeneity, such as changes in pore pressure, pore connectivity, and fluid distribution, were not discussed in detail. The dissociation temperature of MH and the heat transfer process of the sediment emerged as key influencing factors. Figure 6 shows the average temperature change over time for layers A-A, B–B, and C–C within the reactor for Cases 1–4. Additionally, Figure 6 shows the equilibrium dissociation temperatures (T_eq_) of MH in pure water under different pressures. T_eq_ is calculated using the fugacity model given by Li et al.^45^ As shown in Figure 6, in Case 1, the endothermic nature of MH dissociation during depressurization causes a decrease in the temperature within the reactor, with minimum temperatures across different regions approaching the dissociation temperature of MH in pure water, which is consistent with the results of previous studies.^30,34^ In contrast, as the MMT content increases from 10 to 20 wt %, the minimum temperature (point B) in the MMT clay-bearing sediment decreases by approximately 0.30 0.30, and 0.40 K, respectively. This is mainly due to the decrease in free water content in the sediment as the MMT content increases.^31,36^ Consequently, the proportion of MH formed from free water decreases, while that formed from bound water increases. Since MH formed from bound water has a lower dissociation temperature,^39^ these MH dissociate first during DP period, followed by the MH formed from free water. This explains the lower temperature observed at point B in systems with a higher MMT content. In Cases 3 and 4, slight temperature differences in different regions may be attributed to the effects of the MH distribution.

During the CP period, the temperature in undissociated regions initially remains constant and gradually increases to ambient temperature due to continuous heat transfer and MH dissociation. In high MMT content systems, the average temperature increase rate of different layers is significantly lower than that of the sandy system. On the one hand, the inherent thermal conductivity of MMT (1.85 W/m·K) is significantly lower than that of quartz sand (7.70 W/m·K).^46^ As the MMT content increases, the effective thermal conductivity of sandy sediments decreases, reducing the rate of heat transfer between the environment and the reservoir. In terms of MH distribution, as shown in Figure 6, the temperature rise rate in the middle layer of the reactor gradually decreases with increasing MMT content. Due to external heat transfer, the temperature rise rate is slightly higher in the layer closest to the water bath cooling cycle (layers A-A), followed by the lower layer (layers C–C), and the layer B–B in the middle of the reactor is relatively the lowest. This indicates that the MH content in the middle of the reactor increases with the increase of MMT content, leading to a further slower temperature rise rate in the layer B–B, thereby significantly decreasing the dissociation rate of MH. On the other hand, as the depressurization experiment progresses, the amount of dissociated water gradually increases. MMT, which has strong interlayer and surface water absorption capabilities, will further reduce the diffusion rate of CH_4_ gas, hindering the heat and mass transfer during MH dissociation, thereby slowing down the MH dissociation rate.

The results for gas production rates in Section 3.1.2 show that at GR20 (corresponding to the 26th min in Figure 6(b)), the MH dissociation rate in Case 2 is approximately 2.30% higher than that in Case 1. This is because, in the early stages of the depressurization experiment, the low MMT content has a minor effect on the sediment heat transfer process, while the dissociation conditions of MH have a more significant impact. As a result, following the end of the DP period, Case 1 accelerates the heat transfer between the environment and the MH reservoir due to the lower reservoir temperature. However, as low-dissociation-temperature MH dissociates and the reservoir temperature rises to the dissociation temperature of MH in pure water, the heat transfer capacity of the sediment begins to dominate the MH dissociation rate, causing the rate of heat transfer to gradually decrease. Similar phenomena are also observed in Cases 3 and 4: at GR40 and GR60 (corresponding to the 94th and 104th min in Figure 6(c) and 188th and 213th min in Figure 6(d)), the gas production rate of Cases 3 and 4 increases slightly. However, compared to Case 2, the increase in gas production is delayed, mainly because the higher MMT content reduces the heat transfer rate of the sediment, resulting in the delay of gas production acceleration phenomenon in Cases 3 and 4.

### 3.3 Analysis of Resistance Change

The process of MH dissociation is accompanied by an increase in water saturation in the pores, while MH saturation and gas saturation decrease. In general, water has a strong electrical conductivity, whereas MH and methane gas have relatively weak electrical conductivities, especially the CH_4_, which has an electrical conductivity that is only 1/10 that of water. During the DP period, the release of gas significantly affected the electrical conductivity of the MH-bearing sediments, altering the electrical resistance. Therefore, changes in electrical resistance can be used to infer the progress of MH dissociation in different regions.^48^

**Figure 7:**
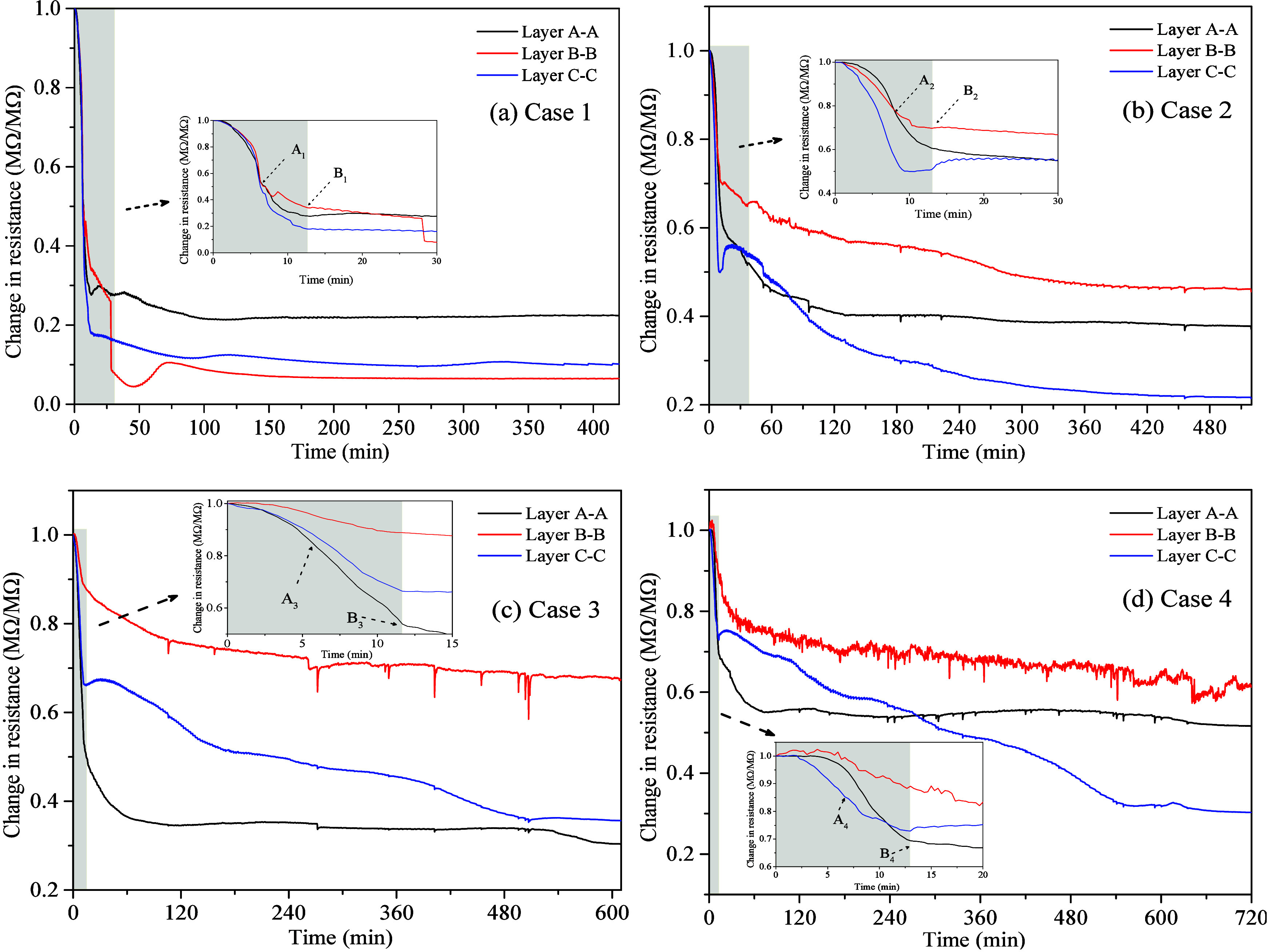
Evolution of the average resistance at layers A-A, B–B, and C–C in experiment Cases 1–4.

Figure 7(a)–(d) shows the changes of average electrical resistance of layers A-A, B–B and C–C in Cases 1–4, respectively. The gray areas represent the DP period, and the white areas represent the CP period. Overall, in each case, the resistance drops more steeply in the DP period than in the CP period. For Case 1, as shown in Figure 7(a), before point A_1_, the average resistance in each region gradually decreases, and the decrease rate gradually accelerates. This may be because, in the early period of DP, the rapid drop in pressure leads to the rapid release of dissolved and trapped CH_4_ gas from pore water, forming free gas bubbles. These bubbles redistribute water, creating more continuous water pathways that increase the conductive paths, resulting in a rapid decrease in resistance. At point A_1_, the average resistances in layers A-A, B–B, and C–C decrease by approximately 46%, 47%, and 50%, respectively. This difference can be attributed to minor variations in the pore structure of each layer. At point B_1_, the resistance decreases further because the MH begins to dissociate and continues to produce dissociated water. At this time, the average resistances in layers A-A, B–B, and C–C decrease by 72%, 66%, and 82%, respectively. This difference may be related to the different distributions of MH in each layer, with the largest resistance decrease in layer C–C, indicating that MH dissociation in this layer is more intense, while the layers A-A and B–B are relatively slower. It is speculated that the MH content in layers C–C is slightly lower than that in layers A-A and B–B. During the CP period, as MH continues to dissociate, the water content in pores gradually increases. Due to the relatively smooth conductive pathways and minimal changes in pore channels, resistance declines proportionally until it reaches a minimal change rate. When the experiment is carried out for about 40 min, significant resistance increases occur in layers B–B. This is due to the proximity of the production well to layer B–B, causing the gas–water balance near the production well to be slightly lagging compared to other layers.

As shown in Figure 7(b)–(d), resistance changes in Cases 2–4 display a trend similar to that in Case 1. The primary difference lies in the more significant resistance differences and decrease rates among different layers. This is due to the strong water absorption capacity of MMT, which weakens the promotion effect of released gas on promoting water flow during MH dissociation, resulting in greater resistance response differences among different layers. In Case 2, at point A_2_, the average resistance of layers A-A, B–B, and C–C decreases by approximately 19%, 20%, and 41%, respectively, with a maximum resistance difference of 22%. At point B_2_, the reductions are about 39%, 30%, and 49%, respectively, with a maximum difference of 19%. At point C_2_, the reductions reach 62%, 54%, and 78%, respectively, with a maximum resistance difference of 24%. In Cases 3 and 4, as the MMT content increases, the resistance differences among different layers further expand, with the maximum differences reaching 36% and 42% at points A, B, and C, respectively. This indicates that higher MMT content leads to greater resistance response differences among different layers, impedes gas release, and limits the improvements in water-phase connectivity.

From Figure 7, it is also evident that in Cases 2–4, the resistance of layers B–B decreases at a slower rate and by a smaller margin at each experimental stage. This can be attributed to the higher MH content in the layer B–B decreases the release rate of dissociated water, delaying the improvement of effective conductive pathways. This result aligns with the MH dissociation process revealed by temperature changes in Section 3.2, further validating the impact of pore structure and MH distribution differences on resistance changes. It should be noted that in Figure 7(c) and (d), the layer B–B curve exhibits some degree of noise, which may be due to that the B–B layer is closer to the production well and the gas–liquid fluctuations near the wellbore may be more pronounced. However, these fluctuations are confined to a specific range and do not significantly affect the overall trend or results.

**Figure 8:**
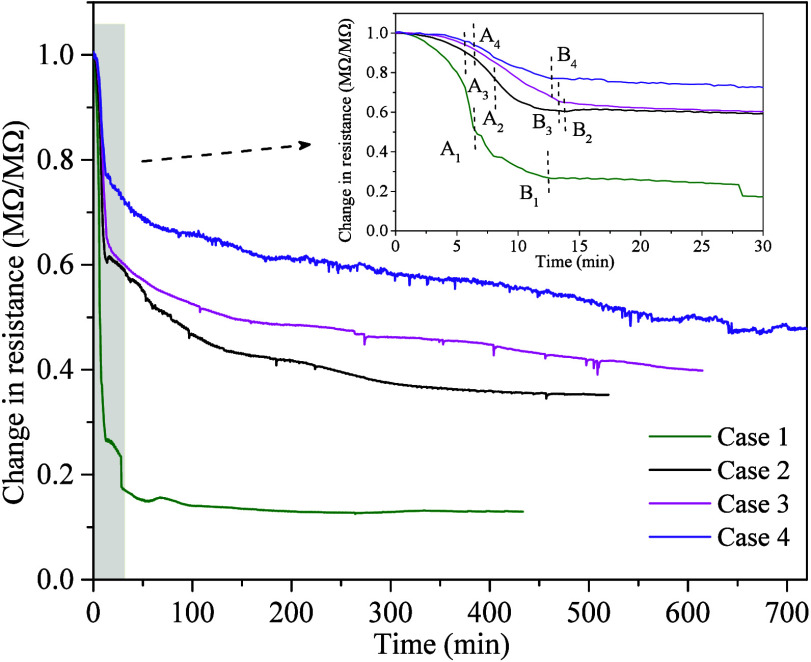
Evolutions of the average resistance in experiments Cases 1–4.

To more clearly illustrate the impact of different MMT contents on resistivity, Figure 8 shows the evolution of average resistance at different layers in Cases 1–4. As shown in Figure 8, in the free gas release period (before point A), with the increase of the MMT content, the average resistance in the reservoir decreases to approximately 49%, 68%, 83%, and 87% of the initial value, respectively. This difference arises from the strong adsorption of pore water by MMT due to its high-water absorption capacity, which limits the promotion effect of gas released from MH dissociation on water phase flow. At the end of the DP phase (point B), the average resistances in the different cases decrease to 27%, 61%, 65%, and 77% of the initial value, respectively. This indicates that higher MMT content in the reservoir hinders the diffusion and redistribution of dissociated water, leading to a smaller decrease in resistance. After point B, due to the significant decrease in the dissociation rate of MH caused by the MMT content, the trend of changes in average resistance and gas recovery rate basically shows a proportional slow decrease. By the end of the experiment, the average resistances in Cases 1–4 decrease to 13%, 35%, 40%, and 47% of the initial value, respectively.

### 3.4 Application

Calcium MMT, a typical layered silicate mineral with high water absorption and specific surface area. When MMT particles undergo a wetting process, swelling occurs between the crystal layers, including lattice expansion and osmotic swelling.^49^ In addition to the expansion of water absorption between crystal layers, the surface of MMT particles also can absorb water molecules via matrix suction,^50^ creating three distinct pore water states: strongly bound, weakly bound, and free water. These hydration states critically influence sediment pore water activity, which in turn affects the stability and dissociation behavior of MH.^34^ During the actual exploitation of NGH, sediment heterogeneity drives multistage dissociation: NGH formed from strongly bound water dissociates first, followed by weakly bound and free water phases. This sequential process may induce dissociation rate fluctuations, potentially destabilizing reservoirs.

In this work, laboratory experiments were conducted under controlled conditions, which may differ from real reservoir environments in several ways. For instance, real reservoir conditions are often more complex and variable with a wider range of pressures, temperatures, and sediment compositions. Therefore, in addition to the results mentioned above, during the actual exploitation of NGH, sediment heterogeneity may introduce challenges, such as variations in pore network connectivity, pore water distribution, and effective permeability, which can change. These factors influence the heat conductivity, heat convection, and mass transfer during depressurization. Moreover, the strong hydrophilicity and low permeability of MMT hinder gas diffusion, impeding heat and mass transfer during NGH dissociation. As a result, the dissociation rate of NGH may be slowed, complicating the extraction process. These factors are crucial in natural reservoir environments and should be addressed in future studies to enhance our understanding of MH dissociation in heterogeneous sediments.

## 4 Conclusion

This study systematically investigated the effect of varying MMT content (0–20 wt %) on MH dissociation. The gas production characteristics, heat transfer behavior, and resistivity variations were analyzed in detail. The main conclusions are as follows:

(1) Compared to sandy sediments, as the MMT mass fraction increases from 10 to 20 wt %, the average MH dissociation rate decreases by approximately 47%–78%, and the cumulative gas production decreases by 0.1% to 16%.
(2) At the end of the depressurization period, the pressure–temperature relationship of MH in sandy sediments is consistent with that of bulk MH in pure water. However, in MMT clay-bearing sediments, excess temperature drops were observed in the depressurization process, with a maximum decrease of ∼0.40 K in sediments containing 20 wt % MMT.
(3) MH dissociation exhibits a transition from distal regions to the central production well. In MMT clay-bearing sediments, the rate of temperature increase in different layers is significantly lower than that of sandy sediments. This is primarily due to the lower thermal conductivity of MMT clay-bearing sediments and the more dispersed distribution of MH.
(4) The resistivity change in sandy sediments is more pronounced than that in MMT clay-bearing sediments. As the MMT content increases from 0 to 20 wt %, the average resistivity decreases by 13%, 35%, 40%, and 47%, respectively, due to the strong water adsorption capacity of MMT, which dispersed water and MH and delayed the formation of a conductive path during MH dissociation. The resistivity results are consistent with temperature analysis, confirming the influence of clay content and MH distribution on resistivity changes.
